# Ground-truthed and high-resolution drone images of the leafy spurge weed plant (*Euphorbia esula*)

**DOI:** 10.1038/s41597-025-05094-6

**Published:** 2025-05-06

**Authors:** Kyle Doherty, Max Gurinas, Erik Samsoe, Charles Casper, Beau Larkin, Philip Ramsey, Brandon Trabucco, Ruslan Salakhutdinov

**Affiliations:** 1 MPG Ranch, Aerial Survey Program, Missoula, MT USA; 2 University of Chicago Laboratory Schools, Chicago, IL USA; 3 Carnegie Mellon University, Department of Machine Learning, Pittsburgh, PN USA

**Keywords:** Invasive species, Restoration ecology

## Abstract

This dataset comprises 1.3 cm resolution aerial images of grasslands in western Montana, USA, captured by a commercial drone. Many scenes contain leafy spurge (*Euphorbia esula*), introduced to North America, now widespread in rangeland ecosystems, which is highly invasive and damaging to crop production and biodiversity. Technicians surveyed 1000 points in the study area, noting spurge presence or absence, and recorded each point’s position with precision global navigation satellite systems. We cropped tiles from an orthomosaic image at these locations. We publicly release these images and metadata as a Hugging Face Dataset, accessible in one line of code. Our aim is to invite the research community to develop classifiers as early warning systems for spurge invasion. We tested classification performance for two contemporary vision models and achieved 0.85 test accuracy. This demonstrates the feasibility yet difficulty of this classification task.

## Background & Summary

Gathering ecological data *in situ* requires expertise in species identification, spatial planning, and haste to capture ephemeral patterns. Consequently, scaling ecological monitoring efforts across larger areas is difficult without supporting technologies such as satellite or drone-based imaging^1^. Agriculture has pioneered these remote sensing approaches to detect and map changes to crop health^2^ and weed plant occurrence^3^. A number of benchmark datasets exist in the agricultural domain with target objectives such as detecting weeds and assessing crop health in high resolution drone imagery^4–8^. Increasingly, remote sensing tools are applied in wildland settings with similar goals: to monitor change in plant communities and mount a management response^9^. Yet, there are few publicly available benchmark datasets containing high resolution imagery of wildlands, and these are biased toward forested areas^10–12^, which represent a minority of terrestrial biomes^13^. While the challenges posed by large-scale ecological monitoring are formidable, this effort is critical to tracking change in natural ecosystems, and the dearth of benchmark datasets in wildlands represents a notable gap.

There are stark differences between remote sensing applications in agricultural and wildland contexts. The diversity of species present in wildland systems is often far greater than those in agricultural contexts^14^. Thus, for tasks such as image classification, discerning target plants from background species can be more difficult, as the background domain is more varied. Furthermore, identifying diagnostic features of target plants, such as flower and leaf morphology, is challenging, requiring botanical expertise in the field. It may be impossible to resolve such features in coarse-grained images, necessitating the use of drone-based platforms that can fly lower and gather fine-grained information^15,16^. The terrain of wildlands is also more complex than in agricultural sites, which are situated in flat areas to facilitate mechanized tillage, seeding, and pest management^17^. As a consequence, gathering data in wildlands is time consuming and costly due to steep terrain, lack of roads, and poor connectivity for navigation systems. Additionally, complex terrain generates varied lighting conditions^18^, which may pose challenges for classifiers^19^. Therefore, applying machine learning solutions to remote sensing of wildland phenomena is inherently more difficult than in agricultural domains because gathering data is costly and the content of images more diverse.

**Fig. 1:**
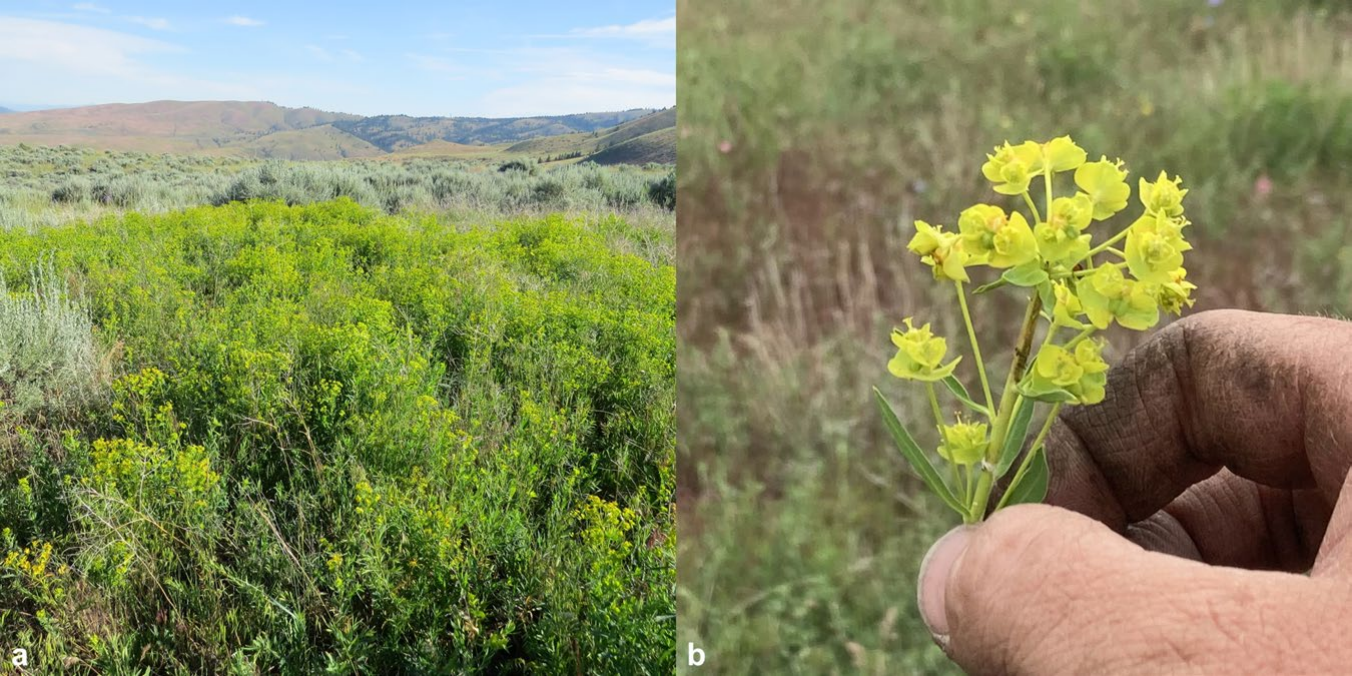
A sagebrush community invaded by leafy spurge (panel **a**) and a closeup of a leafy spurge inflorescence (panel **b**).

One important use-case of remote sensing in wildlands is that of weed plant detection. When an invasive plant invades a natural area, it can cause harm by a variety of mechanisms, including competition with native plants for space and resources^20^, disruption of pollinator services^21^, catalyzing catastrophic wildfire regimes^22,23^, and others. Once established, invasive plants are difficult to remove, requiring expensive monitoring and treatment. Leafy spurge (*Euphorbia esula*; Fig. 1) is an example case introduced to North America in the late 19th century that quickly spread from agricultural areas to wildlands. This noxious weed is avoided by cattle and wild grazing mammals, resulting in economic losses exceeding $100 million in the northern Great Plains^24^. Extensive control methods have been employed to manage its spread^25^, and efforts to monitor expansion are of growing interest. A recent study utilizing 4 m satellite imagery achieved a field-validated accuracy of 0.59^26^, while a prior drone imaging study utilizing 3 cm data achieved an accuracy of 0.78 for flowering spurge plants occupying more than 10% ground cover^27^. Therefore, current evidence suggests higher-resolution imagery can enhance the accuracy of leafy spurge detection.

We gathered high spatial resolution drone imagery of grasslands undergoing ecological restoration in western Montana, USA where leafy spurge has established and is targeted for removal. In parallel we collected ground truth of spurge presence and absence in the field with precision global navigation satellite systems throughout the study region. While the number of drone datasets in the agricultural domain are growing, ours is a unique contribution drawn from a wildlands context. We release and describe these data here for the purpose of advancing leafy spurge detection and management as well as furnishing the machine learning research community with a unique, real-world dataset. We tested two model architectures, including a convolutional neural network and vision transformer, as a basis for validating our data. Please note that a related preprint documenting the value of these data to few and zero-shot research may be found on arXiv^28^.

## Methods

### Drone survey and imagery post-processing

We surveyed the study area on June 12, 2023, during a 4-hour window (from 11:11 to 15:11) with a DJI Mavic 3 M drone. We confirm that all images in this work were captured on private property with the landowner’s permission and drone flights were conducted in strict compliance with FAA regulations and relevant local laws. The drone captured 8241 images at 50 m above ground level across an area of 118 hectares. During the survey there was light wind and sparse cloud cover at 3700 m. We programmed the drone flight using DJI Pilot 2 software provided with the controller such that images overlapped to improve performance of the feature matching algorithm during post-processing, which merges raw images into a single spatially contiguous and georeferenced image, or orthomosaic. The side overlap ratio was 70% and a front overlap ratio was 80% between adjacent images.

The Mavic 3 M is equipped with Real-Time Kinematic (RTK) positioning to enhance the GPS accuracy and ensure centimeter-level precision in the camera position, which was engaged during the flight. The RTK module received position corrections from a Emlid RS2 brand global navigation satellite system receiver (GNSS) set up as a base at the site of flight control. The drone features a 4/3 CMOS image sensor, with a resolution of 20 Megapixels (MP) and operated within an RGB color space. The lens provided a field of view (FOV) of 84° and an equivalent focal length of 24 mm. The ISO range was set between 100 and 6400, with a median shutter speed of 1/640 s. Additional specifications include an actual focal length of 12.29 mm, an aperture of f/2.8, and a minimum exposure time of 1/2,000 s. Images, with dimensions of 5280 × 3956 pixels, varied in size from 9.4 MB to 12.6 MB.

**Fig. 2:**
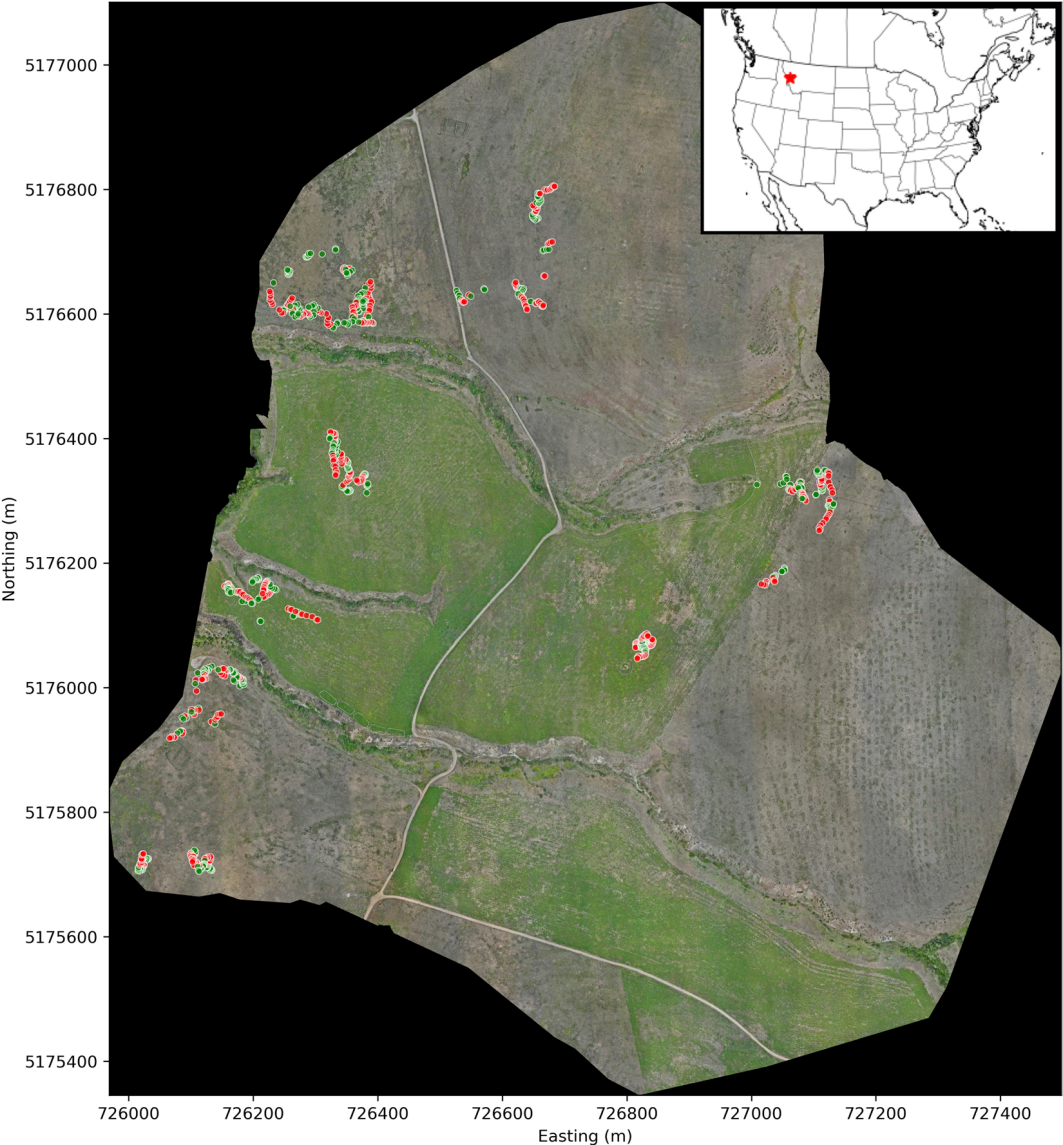
An orthomosaic of the 118-hectare study area at MPG Ranch, Montana, USA where the leafy spurge dataset was gathered. Points are coordinates of field-validated weed presence (green) or absence (red).

We generated an orthomosaic (Fig. 2) with the Drone Deploy post-processing service (https://www.dronedeploy.com). Drone Deploy automates the process of feature matching across overlapping images, correction of geometric distortions, and the generation of a georeferenced product. Prior to surveying, we established 32 ground control points (GCPs; points for which positions were verified with a GNSS reporting sub-centimeter error) across the study area. The locations of GCPs were used during post-processing to further minimize georeferencing error of pixels in the orthomosaic product. The Root Mean Squared Error (RMSE) of GCP position was 7.32 cm after post-processing.

### Leafy spurge ground truth acquisition

**Fig. 3:**
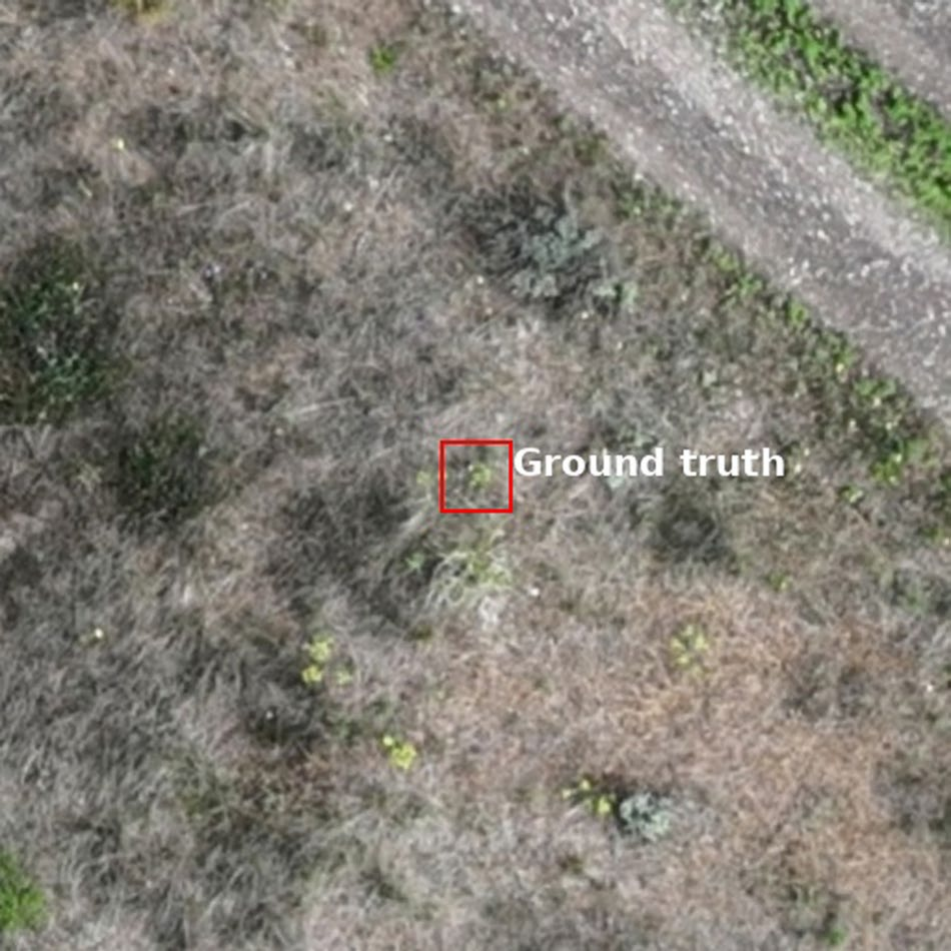
A sample from the orthomosaic where the red box indicates the 0.5 × 0.5 m spatial dimensions of the ground truth.

After surveying our study area technicians visited sites within to gather ground truth of spurge presence and absence. Upon visiting a site, technicians conducted random walks to gather coordinates of spurge presence and absence using an Emlid RS2 GNSS receiver, capable of reporting positions with sub-centimeter accuracy. When a technician encountered a target plant, they would record its position with the GNSS receiver. Technicians also gathered coordinates for spurge absences in this manner. For spurge absence cases, ground truth indicates that no spurge plants were detected in a 0.5 × 0.5 m box centered on the coordinates (Fig. 3). During the walk technicians gathered data until they acquired 50 presences and 50 absences at each site. We visited a total of 10 sites (Point color in Fig. 2), sampling in this manner, and accumulated 500 ground truth points per presence/absence class. While the majority of sites were geographically separated, two sites overlapped due to weather-related time constraints. These were assigned to the same training split (strategy described below) to ensure no data leakage to the test set.

## Data Records

### Dataset access

Our data are hosted publicly as a Hugging Face Dataset^29^, offering immediate access via a Python API. We serve two image sizes corresponding to the 39 × 39 and 1024 × 1024 pixel images as configurations “crop” and “context”, respectively. Additionally, we serve the full unlabeled orthomosaic as configuration “unlabeled.” We authorize use of our data with a Creative Commons Attribution 4.0 International license.

### Image extraction from orthomosaic

**Fig. 4:**
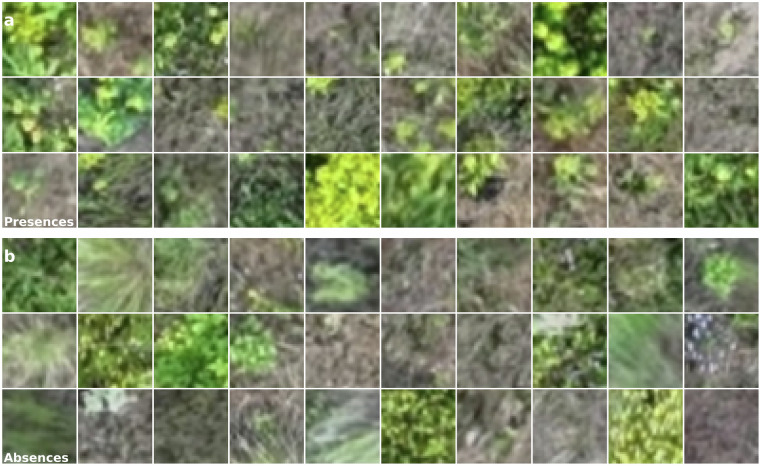
A representative sample of leafy spurge presences (**top; a**) and absences (**bottom; b**).

We extracted a total of 900 images from the orthomosaic which are centered at the coordinates of our ground truth data (Fig. 4). Each instance of ground truth corresponds to 0.5 × 0.5 m, or 39 × 39 pixels (Fig. 3). In addition to the pixels immediately corresponding to the ground truth, we also serve images with greater context extending beyond ground truth bounds, which are 13 × 13 m, or 1024 × 1024 pixels. For each instance we provide metadata (Table 1, such as filename, instance index, geographic and projected coordinates, elevation, time of observation, and geographic sampling cluster (referred to as cluster). Finally, we provide the original orthomosaic, excluding test regions, in tiles. All files associated with the dataset are of geotiff format and are embedded with relevant coordinate reference information.

**Table 1:** Metadata associated with each instance of leafy spurge ground truth.

Feature	Description
file_name	The name of the file (‘.tif’) associated with the data entry.
idx	An index value that serves as a unique identifier for each entry.
label	The categorical label assigned to the entry: 1 (present) or 0 (absent).
longitude	The longitude of the location in decimal degrees (EPSG:4326).
latitude	The latitude of the location in decimal degrees (EPSG:4326).
easting	The UTM Zone 11 N easting coordinate (EPSG:32611).
northing	The UTM Zone 11 N northing coordinate (EPSG:32611).
elevation	The elevation of the location in meters above sea level.
time	A timestamp associated with the data entry (YYYYMMDDHHMMSS).
cluster	The geographic sampling cluster in which the instance was observed.

### Assignment of data splits

We used the geographic separation of our sampling sites as the basis for splitting data for model training and evaluation. We selected eight sites (800 image/label pairs) for the training set and selected the two remaining sites (200 image/label pairs) for test sets. The data from one of these test sites we release with this publication, while we reserve the data from the second site (100 instances) for evaluation of progress at a later date. The intent of establishing test data spatially is to simulate performance on new data gathered from recently invaded areas.

## Technical Validation

In the following sections, we compared the classification performance of two contemporary computer vision models. The intent was to demonstrate the tractability of classifying leafy spurge in our drone imagery with state-of-the-art models. Please find information for reproducing our experiments in the Code Availability section at the end of our work.

### Experiments with computer vision architectures

We evaluated two computer vision architectures for the task of classifying leafy spurge in images:

ResNet50^30^ and DINOv2^31^. ResNet50 is a widely adopted convolutional neural network, while

DINOv2 is a more recent vision transformer-based model. We used pre-trained model checkpoints (facebook/DINOv2-base for DINOv2, microsoft/resnet-50 for ResNet-50) for weight initialization. We pre-processed the leafy spurge images by resizing them to 224 × 224 pixels and then z-scoring their color values using the mean and standard deviation from the ImageNet dataset. This normalization step ensures the color distribution of our images aligns with the data used to pre-train the models, facilitating more effective transfer learning. We conducted experiments with two image sizes from the dataset: 39 × 39 pixels (‘crop’ revision) and 1024 × 1024 pixels (‘context’ revision). The intent of training with larger, 1024-pixel images was to explore if broader context around the ground truth could aid classifier performance, as is reported for larger vision transformer models^32^. To enhance model generalization and mitigate overfitting, we applied the following data augmentation techniques during training with a probability of 0.5: ColorJitter (brightness = 0.8, contrast = 0.7, saturation = 0, hue = 0), RandomHorizontalFlip, RandomVerticalFlip, and RandomRotation (degrees = 90).

We trained for 50 epochs using the Adam optimizer^33^ with a learning rate of 0.0001 and batch size of 32 for both ResNet50 and DINOv2. For the DINOv2 training we applied Low-rank Adaptation^34^ with rank and alpha parameters set to 8. In addition to testing performance on the full dataset, we conducted few-shot experiments, randomly sampling without replacement 1, 2, 4, 8, 16, 32, 64, 128, and 256 examples per class. For each experiment (combination of model and image size, dataset revision, and examples per class), we tested 8 unique seeds (random sample states) to account for variability. For each seed, we split the training set into 80% for training and 20% for validation. We evaluated model performance on both the validation and test sets during training. For our performance metric we calculated the 95% confidence interval of accuracy, defined as the proportion of correctly classified samples. Total compute (computational resources) for these experiments was 248 hours on an internal cluster of 40 Nvidia 2080ti graphics processing units (GPUs). The longest period of training for a single seed was one hour and eighteen minutes, observed with DINOv2 architecture. This experiment is representative of the time it would take to train a production model, and could be replicated with publicly available services, such as GPU-equipped Google Colab runtimes, for less than $10 USD at this time.

### Results from computer vision experiments

**Fig. 5:**
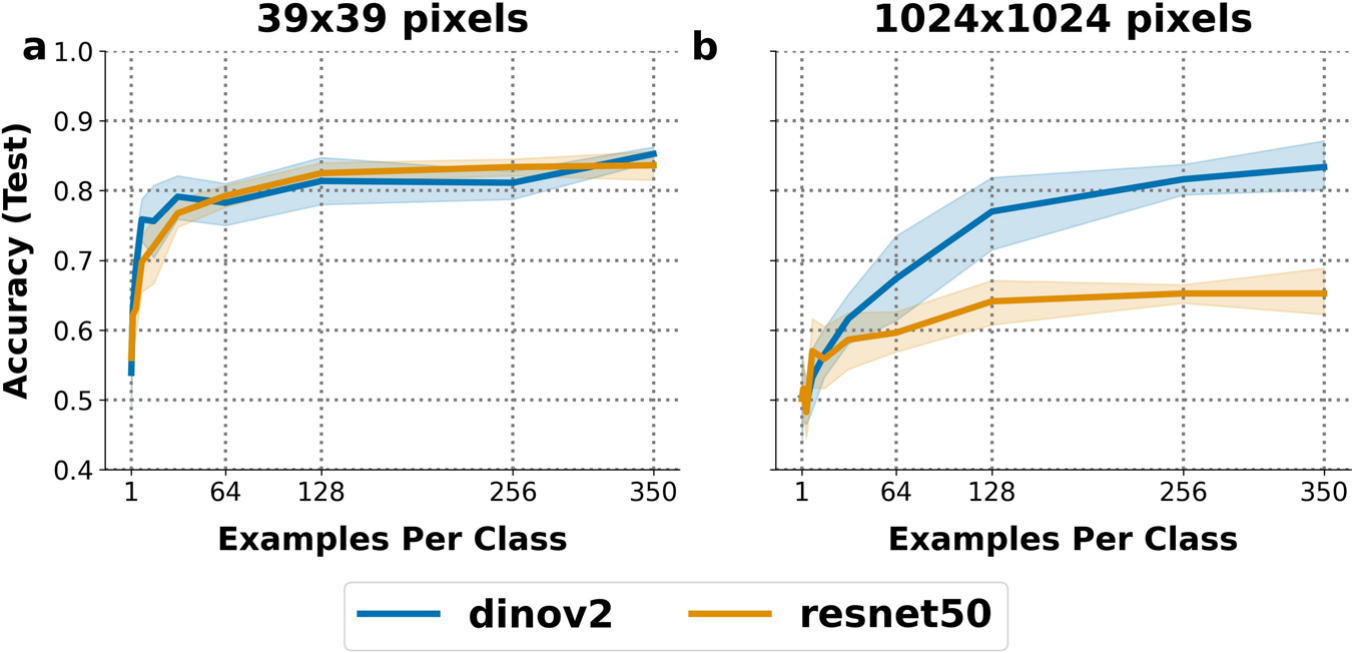
We present classification results on the Leafy Spurge Dataset using ResNet50 (orange) and DINOv2 (blue) architectures. Panel (**a**) shows test set accuracy when training on 39 × 39 pixel images, while panel (**b**) shows results for 1024 × 1024 pixel images. Accuracy is reported across resampled dataset sizes (1 through 256 examples per class) and the full dataset (350 examples per class). Cross-validated means (eight random samples) are represented as lines, with 95% confidence intervals shown as bands.

We found both DINOv2 and ResNet50 architectures suitable for detection of the target plant, leafy spurge, though performance was contingent on image size (Fig. 5). Performance of each model type was similar when trained on the full dataset of smaller images, but ResNet50 could not satisfactorily classify spurge in larger images (1024 × 1024 pixels). Notably, models trained on smaller images (39 × 39) whose dimensions correspond directly to the 0.5 × 0.5 m bounds of ground truth performed better with fewer examples per class than those trained on larger images (1024 × 1024).

## Usage Notes

We hope that those exploring our data will tailor their work to benefit the land management community whose objective is to contain and remove leafy spurge. One primary consideration is that of the spatial scale of spurge predictions. At present, leafy spurge plants are treated with herbicide by a human applicator, who can target individual plants or groups of plants. For these tasks, knowledge of plant presence on the landscape at 0.5 m scale (the scale of a single plant) would be more than adequate for successful treatment. In contrast, pixel-level inference (leaf-scale) offers no practical benefit, as applicators cannot spray at 1.3 cm resolution. Therefore, mapping spurge extents by tiling out the orthomosaic into 39 × 39 pixel images, conducting inference on tiles, and reconstituting the products into a mosaic would be useful for weed management. In addition to labeled images we serve the full unlabeled orthomosaic, excluding test regions. Unsupervised learning on these data might enhance classifier performance and a successful application of this technique could benefit the broader field of remote sensing where vast amounts of unlabeled aerial images are a common condition.

## Data Availability

We provide all Python code for reproducing the experiments in the Technical Validation Section. In a Github repository (https://github.com/leafy-spurge-dataset/leafy-spurge-dataset). For usage documentation of the Hugging Face Datasets API, please refer to the materials found at https://huggingface.co/docs/datasets/en/quickstart.

## References

[1] Turner, W. *et al*. Remote sensing for biodiversity science and conservation. *Trends in Ecology & Evolution* **18**, 306–314 (2003).

[2] Mulla, D. Twenty five years of remote sensing in precision agriculture: Key advances and remaining knowledge gaps. *Biosystems Engineering* **114**, 358–371 (2013).

[3] Lamb, D. & Brown, R. Using remote sensing to identify, map, and monitor the distribution of weeds. *Journal of Agricultural Engineering Research* **78**, 117–125 (2001).

[4] dos Santos Ferreira, A., Pistori, H., Freitas, D. & da Silva, G. Data for: Weed Detection in Soybean Crops Using ConvNets. *Mendeley Data*10.17632/3fmjm7ncc6.2 (2017).

[5] Chiu, M. *et al*. Agriculture-Vision: A Large Aerial Image Database for Agricultural Pattern Analysis. ArXiv preprint 10.48550/arXiv.2001.01306 (2020).

[6] Krestenitis, M. *et al*. CoFly-WeedDB: A UAV image dataset for weed detection and species identification. *Data in Brief* **45**, 108575 (2022). 36131952 10.1016/j.dib.2022.108575 PMC9483728

[7] Genze, N., Ajekwe, R., Grieb, M. & Grimm, D. Deep learning-based early weed segmentation using motion blurred UAV images of sorghum fields. *Mendeley Data*10.17632/4hh45vkp38.4 (2022).

[8] Wildeboer, S. MegaWeeds dataset. *Zenodo*10.5281/zenodo.8077195 (2023).

[9] Pettorelli, N. *et al*. Satellite remote sensing for applied ecologists: opportunities and challenges. *Journal of Applied Ecology* **51**, 839–848 (2014).

[10] Weinstein, B. *et al*. A benchmark dataset for canopy crown detection and delineation in co-registered airborne RGB, LiDAR and hyperspectral imagery from the National Ecological Observation Network. *PLOS Computational Biology* **17**, e1009180 (2021). 34214077 10.1371/journal.pcbi.1009180 PMC8282040

[11] arura uav. UAV Tree Identification - NEW Dataset. *Roboflow Universe* https://universe.roboflow.com/arura-uav/uav-tree-identification-new (2023).

[12] Mowla, M., Asadi, D., Tekeoglu, K., Masum, S. & Rabie, K. UAVs-FFDB: A High-Resolution Dataset for Advancing Forest Fire Detection and Monitoring Using Unmanned Aerial Vehicles (UAVs). *Data in Brief* **55**, 110706 (2024). 39076831 10.1016/j.dib.2024.110706 PMC11284670

[13] Food and Agriculture Organization of the United Nations. *Global Forest Resources Assessment 2020 Key Findings*. Available at: 10.4060/ca8753en (2020).

[14] Phalan, B., Balmford, A., Green, R. & Scharlemann, J. Minimising the harm to biodiversity of producing more food globally. *Food Policy* **36**, S62–S71 (2011).

[15] Gallmann, J. *et al*. Flower Mapping in Grasslands With Drones and Deep Learning. *Frontiers in Plant Science* **12**, 774965 (2022). 35222449 10.3389/fpls.2021.774965 PMC8864122

[16] Amputu, V. *et al*. Unmanned aerial systems accurately map rangeland condition indicators in a dryland savannah. *Ecological Informatics* **75**, 102007 (2023).

[17] Özkan, B., Dengiz, O. & Turan, I. D. Site suitability analysis for potential agricultural land with spatial fuzzy multi-criteria decision analysis in regional scale under semi-arid terrestrial ecosystem. *Scientific Reports* **10**, 22074 (2020). 33328573 10.1038/s41598-020-79105-4 PMC7744537

[18] Corripio, J. G. Vectorial algebra algorithms for calculating terrain parameters from DEMs and the position of the sun for solar radiation modelling in mountainous terrain. *International Journal of Geographical Information Science* **17**, 1–23 (2003).

[19] Rodriguez-Galiano, V. & Chica-Olmo, M. Land cover change analysis of a Mediterranean area in Spain using different sources of data: Multi-seasonal Landsat images, land surface temperature, digital terrain models and texture. *Applied Geography* **35**, 208–218 (2012).

[20] Maron, J. & Marler, M. Field-based competitive impacts between invaders and natives at varying resource supply. *Journal of Ecology* **96**, 1187–1197 (2008).

[21] Pearson, D., Ortega, Y. & Sears, S. Darwin’s naturalization hypothesis up-close: Intermountain grassland invaders differ morphologically and phenologically from native community dominants. *Biological Invasions* **14**, 901–913 (2012).

[22] Balch, J., Bradley, B., D’Antonio, C. & Gomez-Dans, J. Introduced annual grass increases regional fire activity across the arid western USA (1980–2009). *Global Change Biology* **19**, 173–183 (2013). 23504729 10.1111/gcb.12046

[23] Bradley, B. *et al*. Cheatgrass (Bromus tectorum) distribution in the intermountain western United States and its relationship to fire frequency, seasonality, and ignitions. *Biological invasions* **20**, 1493–1506 (2018).

[24] Leistritz, F., Bangsund, D. & Hodur, N. Assessing the economic impact of invasive weeds: the case of leafy spurge (Euphorbia esula). *Weed Technology* 1392–1395 (2004).

[25] Gaskin, J. *et al*. Managing invasive plants on Great Plains grasslands: A discussion of current challenges. *Rangeland Ecology & Management* **78**, 235–249 (2021).

[26] Mattillo, C., Tekiela, D. & Norton, U. Remote mapping of leafy spurge (Euphorbia esula, L.) in Northwestern Colorado. *Frontiers in Remote Sensing* **4** (2023).

[27] Yang, X. *et al*. Mapping flowering leafy spurge infestations in a heterogeneous landscape using unmanned aerial vehicle Red-Green-Blue images and a hybrid classification method. *International Journal of Remote Sensing* **42**, 8930–8951 (2021).

[28] Doherty, K. *et al*. Leafy spurge dataset: Real-world weed classification within aerial drone imagery ArXiv preprint at 10.48550/arXiv.2405.03702 (2024).

[29] Doherty, K. *et al*. Leafy spurge dataset. *Hugging Face*10.57967/hf/2508 (2025).

[30] He, K., Zhang, X., Ren, S. & Sun, J. Deep residual learning for image recognition. *Proceedings of the IEEE Conference on Computer Vision and Pattern Recognition* 770–778 (2016).

[31] Oquab, M. *et al*. DINOv2: Learning Robust Visual Features without Supervision. ArXiv preprint at 10.48550/arXiv.2304.07193 (2023).

[32] McKinzie, B. *et al*. MM1: Methods, Analysis & Insights from Multimodal LLM Pre-training. ArXiv preprint at 10.48550/arXiv.2403.09611 (2024).

[33] Kingma, D. P. & Ba, J. Adam: A Method for Stochastic Optimization. ArXiv preprint at 10.48550/arXiv.1412.6980 (2017).

[34] Hu, E. J. *et al*. LoRA: Low-Rank Adaptation of Large Language Models. ArXiv preprint at 10.48550/arXiv.2106.09685 (2021).

